# Health asset profiles and health indicators among 13- and 15-year-old adolescents

**DOI:** 10.1007/s00038-019-01280-7

**Published:** 2019-07-12

**Authors:** Leena Paakkari, Minna Torppa, Raili Välimaa, Jari Villberg, Kristiina Ojala, Jorma Tynjälä

**Affiliations:** 1 Research Center for Health Promotion, Faculty of Sport and Health Sciences, University of Jyväskylä, P.O. Box 35 (L), 40014 Jyväskylä, Finland; 2 Department of Teacher Education, University of Jyväskylä, Jyväskylä, Finland

**Keywords:** Adolescents, Health assets, Profiles, Health indicator

## Abstract

**Objectives:**

We examined the associations between adolescents’ health assets and various health indicators (smoking, alcohol use, sleep length, physical activity, healthy eating, oral health, self-reported health, multiple health complaints).

**Methods:**

A nationally representative sample was drawn from Finnish-speaking schools, comprising 13- and 15-year-old adolescents (*n* = 3833). The measures taken covered the adolescents’ health assets, which were labelled *Family*-*financial*, *Psychological*, *Family*-*social*, *Friends*-*social*, *School*-*social*, and *Human*. Our analysis applied two-step cluster analysis and multilevel mixed-effects binary logistic regression.

**Results:**

Six asset profiles were identified: ‘Limited in most assets, despite medium affluence’, ‘Mostly average assets, but low affluence’, ‘Mostly average assets, though high affluence’, ‘Mostly above average assets’, ‘Rich in most assets’, and ‘Rich in all assets’. There were significant differences between the profiles in terms of *risk level* and *desirable level* health outcomes.

**Conclusions:**

Adolescents differ in their asset profiles. Having multiple health assets appears to protect adolescents from risky behaviour or poor health, and to promote positive health. There is a need for health initiatives to develop a range of health-protecting and health-promoting assets, rather than focus on only one.

**Electronic supplementary material:**

The online version of this article (10.1007/s00038-019-01280-7) contains supplementary material, which is available to authorized users.

## Introduction

During the last decade, an ever-greater emphasis has been placed on how the circumstances in which people live, grow, develop, and work (i.e. the social determinants of health; Marmot 2005; World Health Organization 2008) contribute to health disparities. Among adolescents, the social determinants of health have been of particular interest, since (as noted by Viner et al. 2012, p. 1643) the effects of these determinants are crucially important for the health of entire populations and for national economic development. With these considerations in mind, in efforts to enhance adolescents’ health, more emphasis has been placed on promoting various health-protecting factors than on merely reducing health risks (Viner et al. 2012). Furthermore, achieving good health would seem to require a comprehensive approach in which externally situated factors would be combined with individual-level factors (Forde and Raine 2008). The aim of understanding health-producing elements at various levels is given special emphasis within *health assets* research (Brooks and Kendall 2013).

In the field of public health and health promotion, the concept of ‘health assets’ has attracted considerable interest in the 21st century. A health asset can be defined as ‘any factor (or resource), which enhances the ability of individuals, groups, communities, populations, social systems and/or institutions to maintain and sustain health and well-being, and to help reduce inequalities’ (Morgan and Ziglio 2007, p. 18). Assets can be both external and internal (Brooks and Kendall 2013; see also Leffert et al. 1998), thus being situated within individuals (e.g. self-esteem, skills) but also within institutions or communities (involving, e.g. family-social support, school connectedness). In fact, from their roots in theories such as salutogenesis, resilience, and social capital (Brooks and Kendall 2013), the central elements of assets-based thinking go back to the 1970s or even earlier. Moreover, similarities can be found with the concept of a ‘developmental asset’, which was introduced in the 1990s as part of a focus on young people’s assets as ‘positive factors that contribute to healthy development’ (Benson et al. 1999, p. 5). This approach has its background in research related to resilience, but also to prevention (Benson et al. 1999). In general, an asset-based approach encompasses the notion that instead of focusing on health risks and on the prevention of diseases, more emphasis should be put on the factors that constitute health and well-being (Benson et al. 1999; Morgan and Ziglio 2007). Deriving from such considerations, the aim has been to find a balance between *assets approaches* and *risk*-*focused (deficit) approaches* (Benson et al. 1999; Morgan and Ziglio 2007), meaning that the factors that society positively desires for its young people should be brought into the agenda, rather than merely the negative indicators that should be avoided (Murphey et al. 2004).

The recommendation to focus on health-producing factors is not new, and there has in fact been considerable research on asset-related factors, including various sorts of capital and their contribution to health (e.g. health-relevant cultural capital, Abel 2008; cultural health capital, Shim 2010). To give a few examples, meta-analyses have indicated a modest but significant association between social capital and health (Gilbert et al. 2013) and well-being (Chu et al. 2010). Chu et al. (2010) found that among adolescents the social support gained from teachers and other school personnel had a stronger association with well-being than that gained from family, friends, or other sources, and further, that perceived support was more important than enacted support. Interestingly, the size of one’s networks has only a minimal relationship with well-being (Chu et al. 2010). Asset research has confirmed the important role of both home and out-of-home settings (e.g. school, neighbourhood) in supporting positive health outcomes, and in protecting from risky behaviours (e.g. Brooks et al. 2012; Fulkerson et al. 2006; García-Moya et al. 2015; Klemera et al. 2017). In addition, family-financial or economic capital has been clearly linked to adolescents’ health. Family affluence explains childhood health disparities (Inchley et al. 2016), and the low socio-economic status of families is associated with poorer health outcomes among adolescents (e.g. Reiss 2013). Overall, as argued by Viner et al. (2012, p. 1647), it seems to be the case that ‘proximal determinants related to social and educational domains [e.g. school, family, peers, and neighbourhood] affect the differences in exposure and vulnerability of young people to health-compromising conditions’.

Research examining the associations between assets and health indicators has mainly focused on a few selected assets. However, few studies have included many assets, to examine whether having more assets makes a larger contribution to adolescents’ health than having fewer assets. One such study is that of Murphey et al. (2004), who found that the number of assets (e.g. academic achievement, connectedness to parents, and feeling valued by the community) was related to engagement in various health behaviours. Having fewer assets was associated with a greater likelihood of engaging in risky behaviours; conversely, having more assets was associated with health-enhancing behaviour. In summarizing the matter, Benson et al. (2011) conclude that assets appear to be additive in nature, and that there may be certain sets of assets that predict certain health outcomes. However, so far not much is known about the range of asset profiles that may exist among adolescents, and how these may be related to various health outcomes. Identification of these asset profiles goes beyond mere consideration of the average experiences of adolescents, allowing exploration of ‘the interindividual variability and complexity that is a hallmark of human growth’ (Benson et al. 2011, p. 218).

In this paper, we use ‘health asset’ as a general term to describe the resources of an individual that may promote or maintain health. Such resources can be expected to be both internal (designated as *Human*, *Psychological*) and external (designated as *School*-*social*, *Friends*-*social*, *Family*-*social*, *Family*-*financial*). This paper thus aims (1) to identify the health asset profiles of 13- and 15-year-old adolescents and (2) to examine the association of the health asset profiles with various health indicators (smoking, alcohol use, length of sleep, physical activity, healthy eating, oral health, self-reported health, multiple health complaints).

## Methods

### Participants

A nationally representative sample from Finnish-speaking schools was collected in Finland during March–May 2014, as part of the *Health Behaviour in School*-*aged Children* (HBSC) study. In total, 3833 adolescents from 359 schools took part in the survey. The schools were chosen from the Finnish school register using a cluster sampling method. Sampling was adjusted to take into account the province within Finland, the type of municipality (urban, semi-urban, rural), and the size of the school (PPS, Proportion Probable Size). The participating classes were selected randomly within each school. Pupils aged 13 and 15 responded during one lesson (45 min) to a standardized paper-and-pen questionnaire. The participants were aware of the confidentiality of the data, and they responded voluntarily and anonymously. The response rate for schools was 68%, while the response rate for the pupils within the participating schools was 85%.

### Measures

#### Health assets

Health assets were divided into six categories designated as *Family*-*financial*, *Psychological*, *Family*-*social*, *Friends*-*social*, *School*-*social*, and *Human* (Table 1). The *Family*-*financial* asset was measured via the Family Affluence Scale (FAS III; Torsheim et al. 2016). The *Psychological* asset was measured by a self-esteem scale (Rosenberg 1965) plus the Body Investment Scale (BIS, subscale on body image; Orbach and Mikulincer 1998). The *Family*-*social* asset was measured by eight scales measuring family routines (eating together, family eating rules, supporting physical activity), general and school-related social support, family communication, quality of family communication, and parental monitoring (Brown et al. 1993). The *Friends*-*social* asset was measured by communication, general support (Zimet et al. 1988), student support, and loneliness. The *School*-*social* asset was measured by four scales, covering teacher support, school-related competence/autonomy, participation, and school perceptions. The *Human* asset was measured by learning difficulties in reading and mathematics, educational aspiration (upper secondary school or vocational school), and health literacy (HLSAC; Paakkari et al. 2016). (Supplementary Table 1 ‘Health assets variables’).

**Table 1:** Health assets: designations, scales, and measures obtained

	Range	Mean	SD
*Family-financial* One scale, six items, e.g. ‘How many computers (PCs, Macs, or laptops) does your family own?’	Low 0–6 Medium 7–9 High 10–13	8.5	1.84
*Psychological* Two scales: *Self*-*esteem scale*, 10 items, e.g. ‘On the whole I am satisfied with myself’. *Emotional investment of the body*, six items, e.g. ‘I am satisfied with my appearance’	27–120	86.2	14.0
*Family-social* Eight scales (35 items): Including *Parental monitoring*, six items, e.g. ‘How much your mother/father really know about what you do with your free time?’	45–153	112.3	17.0
*Friends-social* Four scales: *Communication* (one item), *Loneliness* (one item), General and school-related s*ocial support* (four items), e.g. ‘I can talk about my problems with my friends’	4–37	29.7	6.7
*School-social* Four scales: *Teacher support* (three items), *School-related competence/autonomy* (eight items), *Participation* (three items), *School perceptions* (five items), e.g. ‘Our school is a nice place to be’	27–130	90.7	15.1
*Human* Three scales (19 items): *Learning difficulties in reading and spelling*, *and mathematics* *Educational aspiration:* via question: ‘What do you think you will do when you finish comprehensive school?’ Options: ‘Try to enter to ‘upper secondary school’, ‘double examination’ (i.e. for upper secondary school and vocational school), ‘vocational school or other vocational training’, ‘an apprenticeship’, ‘get a job’, ‘be unemployed’ ‘don’t know’. *Health literacy*: via Health Literacy among School-Aged Children (HLSAC) instrument: 10 items, starting with ‘I am confident that…’, and continuing with items such as ‘When necessary I find health-related information that is easy for me to understand’	1–51 10–40	42.3 32.7	6.0 5.4

Health Behaviour in School-aged Children (HBSC) study, Finland, 2014

#### Health indicators

The health indicators were chosen to give a broad picture of health and to comprise a range of health-enhancing (healthy food, oral health, physical activity, sleep length) and health impairing (alcohol use, smoking) behaviours, and perceived health indicators (health complaints, self-rated health) (Table 2). The *healthy food* index indicated the frequency of eating vegetables and fruits*. Oral health* was measured by the frequency of toothbrushing*. Physical activity*, defined as ‘any activity that increases your heart rate and makes you get out of breath some of the time’, was evaluated with the moderate-to-vigorous physical activity (MVPA) scale (Prochaska et al. 2001). In addition, *sleep length* on school nights and *smoking* was estimated. *Alcohol use* was measured by times being drunk.

**Table 2:** Measures used on health indicators (health behaviour and perceived health indicators) (*n* = 3007–3852)

	Item categories/score	Risk level	Desirable level	Min/Max score	Mean	SD	Crh α
Oral health *How often do you brush your teeth?*	1 = more than once a day to 5 = never	< 2 times/day	> 1 times/day	1/5	1.49	0.69	–
Healthy food Two items: Frequency of eating fruits/vegetables *How many times do you usually eat…?*	1 = never to 7 = every day, more than once	0–4 times/week	≥ 7 times/week	0/14	8.90	2.58	0.74
Alcohol use *During your lifetime*, *have you ever had so much alcohol that you were really drunk?*	Never to more than 10 times	≥ 2 times	Never				
Physical activity *Over the past 7* *days*, *on how many days were you physically active for a total of at least 60* *min per day?*	0 to 7 days	0–2 days/week	7 days/week	0/7	4.50	1.92	–
Smoking *How often do you smoke tobacco at present?*	1 = every day to 4 = I don’t smoke	Weekly or more often	Do not smoke	1/4	3.72	0.77	–
Sleep length *Difference between bedtime and wake*-*up time*	Hours	≤ 7 h	≥ 8.5 h	3/11	8.09	0.99	–
Self-rated health *Would you say your health is……?*	1 = excellent to 4 = poor	Fair or poor	Excellent or good	1/4	1.95	0.67	–
Multiple health complaints *In the last 6* *months: how often have you had the following….? headache*, *stomach*-*ache*, *backache*, *feeling low*, *irritability or bad temper*, *feeling nervous*, *difficulties in getting to sleep*, *feeling dizzy*	1 = almost daily to 5 = more seldom or never	≥ 2 symptoms more than once a week	≤ 2 symptoms once a month or seldom/never	0/8	2.81	2.33	0.85

The Health Behaviour in School-aged Children (HBSC) study, Finland, 2014

Perceived health was rated by multiple *self*-*rated health complaints* over the last six months, using the HBSC-SCL symptom checklist (Ravens-Sieberer et al. 2008) and by s*elf*-*rated health* (SRH), which was measured by a single item (Haugland et al. 2001). Health indicators were divided into ‘risk level’ and ‘desirable level’ based on the risk levels used in the international report on HBSC results (Inchley et al. 2016) and on for example national recommendations (e.g. recommendation on physical activity for health).

### Statistical analysis

Two-step cluster analysis was used to identify asset profiles, using categorical and continuous variables. This classification method builds profiles based on similarities in assets. It aims to maximize between-profile variance while minimizing within-profile variance (see Ketchen and Shook 1996). In identifying the cluster solution, we allowed the analysis method to automatically determine the number of the clusters (using Schwarz’s Bayesian Information Criterion) and also tested various fixed numbers of clusters. In deciding on the final cluster solution, the following issues were taken into account: cluster quality (‘fair’ or better > 0.2), the predictor importance of assets in estimating the cluster model, the size of the clusters, and the interpretability. A one-way analysis of variance with Dunnett’s T3 pairwise test (SPSS 24) was utilized to compare the clusters. Multilevel mixed-effects binary logistic regression analyses were applied, using Stata (version 15) to test whether the clusters differed from each other in health behaviours and health indicators, and whether the results varied by school. The results are presented with the age group and gender adjusted.

## Results

### Identification of the health asset profiles

**Fig. 1:**
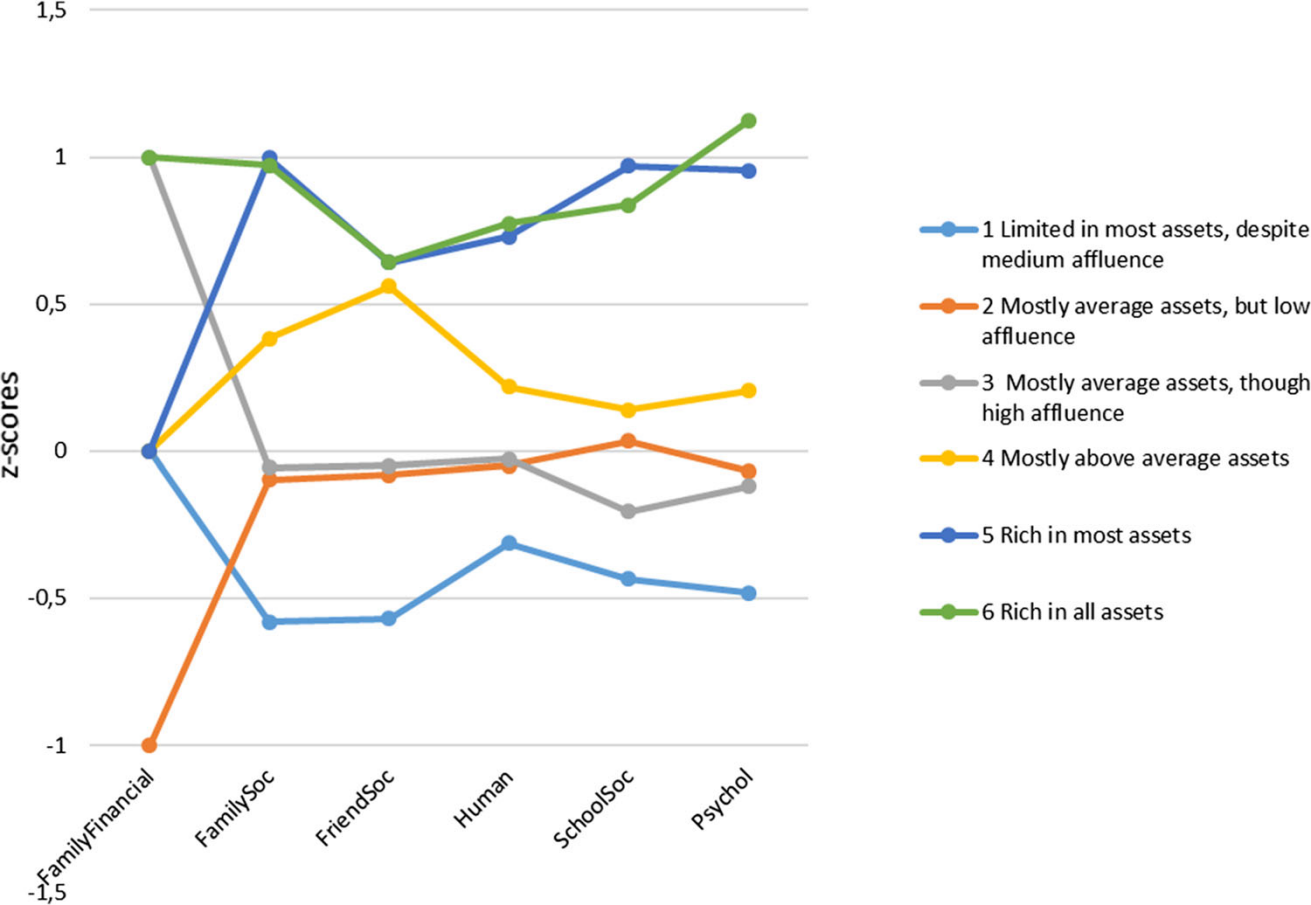
Asset profiles with standardized scores (M = 0, SD = 1), except for Family-financial, where the raw scores 1, 2, 3 were changed to − 1, 0, 1). Health Behaviour in School-aged Children (HBSC) study, Finland, 2014

Having critically examined models from two to seven clusters, we ended up with a six-cluster model. The cluster solution had an average silhouette of 0.3, which suggests a fair fit with the data. As predictors of the cluster model (i.e. having a predictor importance value ranging from 0 to 1; IBM 2012), the assets were ranked thus: *Family*-*social* (1.0) and *Family*- *financial* (1.0), followed by *Psychological* (0.92), *Friends*-*social* (0.72), *School*-*social* (0.71) and *Human* (0.48). Figure 1 and Table 3 present the means and standard deviations for the assets in each cluster, and the statistical differences between the mean values of the clusters.

**Table 3:** Descriptive statistics and cluster comparisons for the asset measures

	1 Limited in most assets, despite medium affluence		2 Mostly average assets, but low affluence		3 Mostly average assets, though high affluence		4 Mostly above average assets		5 Rich in most assets		6 Rich in all assets		F(5, 3645)	Pairwise (Dunnett-T3)
	M	SD	M	SD	M	SD	M	SD	M	SD	M	SD		
Family-social	− 0.58	0.81	− 0.10	0.91	− 0.05	0.83	0.38	0.50	1.00	0.45	0.97	0.43	416.26***	2 = 3, 5 = 6, others differ^1^
Friends-social	− 0.57	0.99	− 0.08	1.01	− 0.05	0.97	0.56	0.39	0.64	0.49	0.64	0.50	239.29***	2 = 3, 4 = 5 = 6, others differ
Human	− 0.31	0.99	− 0.05	0.91	− 0.03	0.92	0.22	0.56	0.73	0.34	0.78	0.30	150.55***	2 = 3, 5 = 6, others differ
School-social	− 0.43	0.91	0.03	0.94	− 0.20	0.92	0.14	0.61	0.97	0.67	0.84	0.75	233.17***	2 = 4, 5 = 6, others differ
Psychological	− 0.48	0.85	− 0.07	1.03	− 0.12	0.78	0.20	0.63	0.96	0.64	1.12	0.53	314.23***	2 = 3, others differ

Health Behaviour in School-aged Children (HBSC) study, Finland, 2014

***p < 0.001

^1^*p* < 0.05

The largest cluster (28.8% of the sample, *n* = 1061) was referred to as ‘Limited in most assets, despite medium affluence’. This was constituted from pupils who had medium family affluence, but who otherwise had fewer health assets than pupils in the other clusters (Table 3). The clusters ‘Mostly average assets, but low affluence’ (13.2%, *n* = 488) and ‘Mostly average assets, though high affluence’ (21.4%, *n* = 790) moved in a roughly parallel direction, but showed statistically significant differences in terms of *Family*-*financial* and *School*-*social* assets. The cluster designated ‘Mostly above average assets’ (18.2%, *n* = 673) included pupils whose assets (other than *Family*-*financial*) were on average higher than those in clusters 1–3. The clusters ‘Rich in most assets’ (11.5%, *n* = 426) and ‘Rich in all assets’ (6.8%, *n* = 252) included pupils with the highest asset measures overall. Except for differences in *Family*-*financial* and *Psychological* assets, there were no statistically significant differences between these two clusters (Table 3).

### Comparing health indicators between clusters

The proportions of adolescents who were categorized as being in a *risk* group, in terms of the various health indicators included in this study, varied from 4.3% (healthy food) to 40.2% (oral health). The proportions of adolescents who reached the *desirable* level varied from 21.3% (physical activity) to 86.3% (smoking). Table 4 reports, for each asset cluster, the percentage of pupils falling into the *risk* group, and those reaching the *desirable* level.

**Table 4:** Proportions of adolescents (per given cluster) falling within the *risk* and *desirable* health categories for each health indicator

	Limited in most assets, despite medium affluence	Mostly average assets, but low affluence	Mostly average assets, though high affluence	Mostly above average assets	Rich in most assets	Rich in all assets	Total
**Oral health**							
Brushing teeth < 2 times a day (*risk*)	49.86% (*n* = 524)	47.61% (*n* = 229)	35.47% (*n* = 277)	37.54% (*n* = 250)	29.12% (*n* = 122)	25.20% (*n* = 63)	40.68% (*n* = 1465)
Brushing teeth > 1 time per day (*desirable*)	50.14% (*n* = 527)	52.39% (*n* = 252)	64.53% (*n* = 504)	62.46% (*n* = 416)	70.88% (*n* = 297)	74.80% (*n* = 187)	59.84% (*n* = 2183)
**Healthy food**							
0–4 times/week (*risk*)	6.6% (*n* = 69)	6.0% (*n* = 29)	3.6% (*n* = 28)	3.2% (*n* = 21)	2.1% (*n* = 9)	0.8% (*n* = 2)	4.3% (*n* = 158)
≥ 7 times/week (*desirable*)	77.19% (*n* = 812)	79.50% (*n* = 384)	85.79% (*n* = 670)	87.24% (*n* = 581)	91.17% (*n* = 382)	94.40% (*n* = 236)	84.95% (3065)
**Alcohol use (being drunk)**							
≥ 2 times (*risk*)	21.8% (*n* = 225)	13.4% (*n* = 64)	21.6% (*n* = 166)	18.1% (*n* = 120)	9.5% (*n* = 40)	12.0% (*n* = 30)	17.9% (*n* = 645)
Never (*desirable*)	68.57% (*n* = 707)	78.71% (*n* = 377)	69.22% (*n* = 533)	74.55% (*n* = 495)	84.01% (*n* = 352)	81.60% (*n* = 204)	73.84% (*n* = 2668)
**Physical activity**							
0–2 days/week (*risk*)	23.2% (*n* = 243)	17.0% (*n* = 82)	13.3% (*n* = 103)	15.7% (*n* = 104)	8.1% (*n* = 34)	8.4% (*n* = 21)	16.1% (*n* = 587)
7 days/week (*desirable*)	17.4% (182)	19.13% (*n* = 92)	21.42% (*n* = 166)	21.08% (*n* = 140)	26.25% (*n* = 110)	34% (*n* = 85)	21.32% (*n* = 775)
**Smoking**							
Weekly/more often (*risk*)	12.4% (*n* = 130)	11.2% (*n* = 411)	7.9% (*n* = 61)	7.8% (*n* = 61)	3.1% (*n* = 13)	0.4% (*n* = 1)	8.5% (*n* = 311)
Do not smoke (*desirable*)	80.69% (*n* = 848)	85.27% (*n* = 411)	86.19% (*n* = 668)	87.54% (*n* = 583)	94.50% (*n* = 418)	95.60% (*n* = 239)	86.33% (*n* = 3144)
**Sleep length**							
≤ 7 h (*risk*)	20.0% (*n* = 209)	17.2% (*n* = 82)	21.5% (*n* = 166)	13.6% (*n* = 90)	7.4% (*n* = 31)	10.0% (*n* = 25)	16.6% (*n* = 603)
≥ 8.5 h (*desirable*)	41.51% (*n* = 433)	48.95% (*n* = 233)	38.60% (*n* = 298)	49.40% (*n* = 328)	63.48% (*n* = 266)	54.22% (*n* = 135)	46.73% (*n* = 1693)
**Self-rated health**							
Fair/Poor (*risk*)	26.41% (*n* = 276)	19.04% (*n* = 91)	15.74% (*n* = 122)	9.17% (*n* = 61)	4.08% (*n* = 17)	3.61% (*n* = 9)	15.87% (*n* = 576)
Good/Excellent (*desirable*)	73.59% (*n* = 769)	80.96% (*n* = 387)	84.26% (*n* = 653)	90.83% (*n* = 604)	95.92% (*n* = 400)	96.39% (*n* = 240)	84.13% (*n* = 3053)
**Subjective health complaints**							
> 2 symptoms more than once a week (*risk*)	39.3% (*n* = 412)	34.7% (*n* = 167)	34.3% (*n* = 267)	21.1% (*n* = 140)	11.0% (*n* = 46)	18.0% (*n* = 45)	29.6% (*n* = 1077)
≤2 symptoms Once a month or Seldom/Never (*desirable*)	28.4% (*n* = 298)	36.4% (*n* = 175)	28.5% (*n* = 222)	40.5% (*n* = 269)	59.7% (*n* = 222)	52.0% (*n* = 130)	38.9% (*n* = 1344)

Health Behaviour in School-aged Children (HBSC) study, Finland, 2014

In the multilevel logistic regression analyses to determine whether the asset clusters were linked to health indicators (risky or desirable) the ‘Limited in most assets, despite medium affluence’ cluster was used as a reference group to which all other clusters were compared. Table 5 reports the odds ratios, the 95% CIs, and the *p* values. The multi-level analysis indicated that there were significant differences between schools. The inter-school variance (variance partition coefficient, VPC) ranged from 1.9 to 12.4% of the total variance, depending on the indicator under scrutiny, and its effect was therefore controlled in the analyses.

**Table 5:** Risk behaviours and health indicators by cluster

		Limited in most assets, despite medium affluence	Mostly average assets, but low affluence	Mostly average assets, though high affluence	Mostly above average assets	Rich in most assets	Rich in all assets	School level factor	
**Oral health**									
Brushing teeth < 2 times a day (*risk*)	OR 95% CI *p* value	1.00	0.96 0.76–1.21 0.736	0.59 0.48–0.73 < 0.001	0.64 0.51–0.79 < 0.001	0.40 0.30–0.52 < 0.001	0.31 0.22–0.43 < 0.001	Variance 95% CI	0.21 0.13–0.33
Brushing teeth > 1 time per day (*desirable*)	OR 95% CI *p* value	1.00	1.04 0.82–1.32 0.74	1.68 1.37– 2.07 < 0.001	1.57 1.26–1.94 < 0.001	2.52 1.94–3.28 < 0.001	3.22 2.30–4.50 < 0.001	Variance 95% CI	0.21 0.13–0.33
**Healthy food**									
0–4 times a week (*risk*)	OR 95% CI *p* value	1.00	0.96 0.61–1.52 0.874	0.58 0.37–0.92 0.020	0.50 0.30–0.83 0.007	0.33 0.16–0.66 0.002	0.11 0.03–0.47 0.003	Variance 95% CI	0.06 0.00–5.75
≥ 7 times a week (*desirable*)	OR 95% CI *p* value	1.00	1.11 0.84–1.46 0.450	1.65 1.27–2.13 < 0.001	1.93 1.46–2.55 < 0.001	3.06 2.10–4.46 < 0.001	5.13 2.91–9.06 < 0.001	Variance 95% CI	0.13 0.06–0.35
**Alcohol use**									
Being drunk: ≥2 times (*risk*)	OR 95% CI *p* value	1.00	0.56 0.40–0.77 < 0.001	1.14 0.89–1.47 0.310	0.84 0.64–1.10 0.202	0.39 0.26–0.56 < 0.001	0.52 0.33–0.81 0.004	Variance 95% CI	0.22 0.12–0.42
Never (*desirable*)	OR 95% CI *p* value	1.00	1.66 1.26–2.20 < 0.001	0.92 0.73–1.15 0.467	1.29 1.01–1.64 0.037	2.38 1.74–3.27 < 0.001	1.91 1.31–2.79 0.001	Variance 95% CI	0.21 0.12–0.37
**Physical activity**									
0–2 days a week (*risk*)	OR 95% CI *p* value	1.00	0.68 0.51–0.90 0.007	0.50 0.39–0.65 < 0.001	0.60 0.46–0.78 < 0.001	0.29 0.20–0.43 < 0.001	0.31 0.19–0.50 < 0.001	Variance 95% CI	0.12 0.05–0.32
7 days a week (*desirable*)	OR 95% CI *p* value	1.00	1.11 0.84–1.48 0.448	1.30 1.02–1.66 0.031	1.29 1.00–1.65 0.051	1.68 1.27–2.22 < 0.001	2.37 1.73–3.26 < 0.001	Variance 95% CI	0.10 0.04 –0.26
**Smoking**									
Weekly/More often (*risk*)	OR 95% CI *p* value	1.00	0.93 0.65–1.34 0.702	0.68 0.48–0.95 0.026	0.62 0.43–0.88 0.008	0.24 0.13–0.43 < 0.001	0.03 0.00–0.23 0.001	Variance 95% CI	0.45 0.25–0.82
Do not smoke (*desirable*)	OR 95% CI *p* value	1.00	1.33 0.97–1.83 0.074	1.36 1.03–1.79 0.029	1.62 1.21–2.18 0.001	4.15 2.60–6.62 < 0.001	4.51 2.37–8.59 < 0.001	Variance 95% CI	0.47 0.29–0.74
**Sleep length**									
< 7 h (*risk*)	OR 95% CI *p* value	1.00	0.84 0.63–1.12 0.232	1.11 0.87–1.40 0.398	0.63 0.48–0.83 0.001	0.32 0.22–0.48 < 0.001	0.45 0.29–0.71 0.001	Variance 95% CI	0.11 0.04–0.33
≥ 8.5 h (*desirable*)	OR 95% CI *p* value	1.00	1.35 1.07–1.70 0.011	0.87 0.71–1.07 0.187	1.39 1.13–1.71 0.002	2.46 1.92–3.15 < 0.001	1.68 1.25–2.26 0.001	Variance 95% CI	0.15 0.09–0.27
**Self-rated health**									
Fair/poor (*risk*)	OR 95% CI *p* value	1.00	0.65 0.50–0.86 0.002	0.52 0.40–0.66 < 0.001	0.28 0.21–0.38 < 0.001	0.12 0.07–0.19 < 0.001	0.10 0.05–0.21 < 0.001	Variance 95% CI	0.14 0.06–0.34
Good/excellent (*desirable*)	OR 95% CI *p* value	1.00	1.53 1.16–2.01 0.002	1.94 1.52–2.47 < 0.001	3.59 2.66–4.86 < 0.001	8.65 5.20–14.40 < 0.001	9.68 4.88–19.19 < 0.001	Variance 95% CI	0.14 0.06–0.34
**Subjective health complaints**									
≥ 2 symptoms more than once a week (*risk*)	OR 95% CI *p* value	1.00	0.76 0.62–0.99 0.044	0.74 0.60–0.91 0.003	0.36 0.29–0.46 < 0.001	0.17 0.12–0.24 < 0.001	0.32 0.22–0.46 < 0.001	Variance 95% CI	0.09 0.04–0.23
≤ 2 symptoms once a month or Seldom/Never (*desirable*)	OR 95% CI *p* value	1.00	1.51 1.19–1.92 0.001	1.08 0.87–1.33 0.492	1.87 1.51–2.31 <0.001	4.17 3.25–5.34 <0.001	2.90 2.16–3.90 <0.001	Variance 95% CI	0.08 0.04–0.20

Mixed effect binary logistic regression models per cluster: odds ratios (OR), 95% confidence intervals (CI), and variance of the school level factor. *The first cluster (Limited in most assets, despite medium affluence) is the reference category. Health Behaviour in School-aged Children (HBSC) study, Finland, 2014

*Adjusted for gender and age group. Reference group marked as 1.00 throughout

The findings suggested that the adolescents in the ‘Limited in most assets, despite medium affluence’ cluster reported less physical activity, poorer self-rated health, and more symptoms than all the other clusters. For example, in the ‘Rich in all assets’ cluster, there was only one-third of the risk of being in the risk group for physical activity (0–2 days a week), as compared to the reference group. In oral health, healthy food, and smoking, all the clusters (with one exception, namely the ‘Mostly average assets, though high affluence’ cluster) indicated a lower likelihood of risk behaviour, or of having frequent and many symptoms, or of reporting fair or poor health, compared to the reference group. In fact, the ‘Mostly average assets, though high affluence’ cluster differed least from the reference group in all health behaviours and health indicators (Table 5). The cluster differences at the desirable end of the scales were here similar to those at the risk end.

## Discussion

Among the adolescents under study, six profiles were identified, labelled as ‘Limited in most assets despite medium affluence’, ‘Mostly average assets, but low affluence’, ‘Mostly average assets though high affluence’, ‘Mostly above average assets’, ‘Rich in most assets’, and ‘Rich in all assets’. There were statistically significant differences between the health asset profiles in terms of their associated health indicators (both regarding the risk level and the desirable level). The largest profile encompassed those moderately affluent adolescents who had below average levels of *Family*-*social*, *School-social*, *Friends*-*social*, *Psychological*, and *Human* assets (thus being ‘Limited in most assets despite medium affluence’). The pupils in this asset profile reported the lowest frequency of physical activity and the lowest self-rated health (fair/poor). They also reported the highest frequency of having multiple health complaints more than once a week. On the other hand, those adolescents who were rich in most/all assets were systematically the least likely to belong to a risk group, in terms of all measured health indicators. This is in line with a previously reported finding, that the more the assets, the smaller the risk of belonging to the group of adolescents with unfavourable health indicators (Murphey et al. 2004).

According to our findings, having numerous assets was also associated with a greater likelihood of belonging to a group of adolescents with several favourable health indicators. Compared to the ‘Limited in most assets, despite medium affluence’ group, the adolescents in the ‘Rich in most assets’ or ‘Rich in all assets’ categories were systematically more likely to belong to a group with all desirable health indicators. Hence, having several health assets appears to *protect from* negative health indicators and also to *promote* good health.

However, though having more assets appeared to be better in most cases, there were only three out of eight health indicators (physical activity, self-rated health, and multiple health complaints) for which the risk was significantly higher in the reference profile (‘Limited in most assets despite medium affluence’) than in the other profiles. Moreover, in relation to favourable health indicators, the reference group did not systematically report desirable health outcomes less often for all the indicators. This may indicate that certain groups of assets do indeed predict certain health outcomes (Benson et al. 2011). It may also be that some individual assets may be more crucial than other assets in protecting from certain kinds of risky behaviour or poorer health, and in promoting certain favourable health outcomes. An interesting finding was that high family affluence alone did not protect adolescents from health-harming behaviours, or from low self-reported health. Neither did low family affluence alone automatically expose a child to unfavourable health outcomes. The findings allow us to suggest that even although socio-economic inequalities in child health are a major problem, and although in many countries the gap between socio-economic groups has deepened with regard to adolescent health (Elgar et al. 2015), efforts to promote other health protective factors—which are often immaterial—may tend to even out such a financially rooted gap. Indeed, the role of immaterial resources has been highlighted previously in health disparity discussions. Thus, Mackenbach (2012, p. 766) has noted that the policies of welfare states have ‘contributed to making an “affluent lifestyle” widely affordable’; nevertheless, they may paradoxically ‘have contributed to a widening of health inequalities’ by overlooking the importance of immaterial resources.

The findings underline the importance of identifying those children who lack various health-protecting and health-promoting factors, and who are thus more vulnerable to poorer health outcomes. A longitudinal study by Frech (2012; see also Cheney et al. 2015) indicated that the resources possessed by adolescents, such as social support from family, school, and friends, plus various psychosocial factors, had ‘a persistent role in promoting healthy behaviour engagement during the transition to adulthood’ (p. 67). Although the present study produced important information on various asset profiles and on their associations with several health indicators, it did not focus on individuals’ genuine possibilities and abilities to make health-promoting choices going beyond the health assets they possess. As discussed by Abel and Frohlich (2012) in the field of capital research, possession of health resources may not in itself decrease disparities; inequalities also exist in options to make healthy choices, and in people’s abilities to actively use various sets of health resources in ways that promote health.

Due to the nature of the constructs measured, conclusions cannot be drawn regarding the effects of unique factors (e.g. loneliness) or of broader assets (e.g. Friends-social) on specific health indicators. Furthermore, additional research is needed on those health indicators that could not be explained by the number of health assets possessed, with efforts to identify the interplay between the unique factors that may both increase and decrease health inequalities—bearing in mind that one individual’s asset may not necessarily be everyone’s asset (cf. De Clercq et al. 2016). Indeed, more discussion is needed on the complexity of interplay between the resources and health behaviour plus health, as has been done in the capital field (e.g. Kawachi and Berkman 2014). Moreover, health assets approach in general has been criticized that while the focus has been on the determinants of health rather than illness and on what people have rather than lack, several complex issues have been avoided such as the discussion about the social gradient of health as well as how health has been shaped by those with power and wealth (Friedli 2013). Critical examination of these could develop the field further. There is also a need for more research employing objective health measures instead of purely subjective ones. Self-reported data are always susceptible to the tendency to provide socially desirable responses, and this can apply also to reports on health behaviours.

To conclude, adolescents differ in their asset profiles. Having multiple health assets appears to protect adolescents from risky behaviour and poor health, and to promote the acquisition of favourable health outcomes. This being so, the development of health assets may contribute to reducing differences in health (Morgan and Ziglio 2007). Furthermore, although the development of even a single asset may bring about positive changes (Murphey et al. 2004), adolescents could benefit more from health promotion and public health initiatives that would support them in acquiring a broad range of assets capable of protecting and promoting health.

## Electronic supplementary material

Below is the link to the electronic supplementary material.

Supplementary material 1 (DOCX 33 kb)
