# Supplementary material for: Health asset profiles and health indicators among 13- and 15-year-old adolescents
Source: Int J Public Health. 2019 Jul 12;64(9):1301–11. doi: 10.1007/s00038-019-01280-7 (PMC6868109; doi:10.1007/s00038-019-01280-7)
Supplement: Supplementary file 1 — Supplementary material 1 (DOCX 33 kb) [file 38_2019_1280_MOESM1_ESM.docx]

***International Journal of Public Health***

Health asset profiles and health indicators among 13- and 15-year-old adolescents

|  | **Question, items and response categories** | **Reference** |
| --- | --- | --- |
| FAMILY FINANCIAL |  |  |
| Family affluence (FAS III) | *Does your family own a car (a passenger car, a van or a lorry)?* ‘none’, ‘one’, ‘two or more’*;*  *Do you have a bedroom for your own? ‘*no’, ‘yes’*;*  *How many computers does your family have (including laptops and tablets, but not game consoles and smartphones)?* ‘none’, ‘one’, ‘two’, ‘more than two’;  *How many bathrooms do you have in your home?* ‘none’, ‘one’, ‘two’, ‘more than two’*;*  *Do you have a dishwasher in your home? ‘*no’, ‘yes’;  *How many times did you travel abroad for holidays with your family last year? ‘*not at all’, ‘once’, ‘twice’, ’more than twice’ | Torsheim T, Cavallo F, Levin KA, Schnohr C, Mazur J, Niclasen B, Currie C (2016) FAS Development Study Group. Psychometric validation of the revised family affluence scale: a latent variable approach. Child Indic Res S1;9(3):771-84 |
| PSYCHOLOGICAL |  |  |
| Self-esteem | *Below is a list of statements dealing with your general feelings about yourself. Please indicate how strongly you agree or disagree with each statement*. (‘strongly agree’, ‘agree’, ‘disagree’, ‘strongly disagree’)  On the whole, I am satisfied with myself;  At times I think I am no good at all;  I feel that I have a number of good qualities;  I am able to do things as well as most other people;  I feel I do not have much to be proud of;  I certainly feel useless at times;  I feel that I'm a person of worth, at least on an equal plane with others;  I wish I could have more respect for myself;  All in all, I am inclined to feel that I am a failure;  I take a positive attitude toward myself | Rosenberg M (1965) Society and the adolescent self-image. Princeton university press |
| Body Investment Scale | *Here are some statements about one’s feelings of his/her body. There are no right or wrong answers. We would like to know how you ​feel about your body. Please tick a box to show how much you agree or disagree with each one. Please tick one box for each line.* (‘strongly disagree’, ‘disagree’, ‘neither agree not disagree’, ‘agree’, ‘strongly agree’)  I am frustrated with my physical appearance;  I am satisfied with my physical appearance;  I hate my body;  I feel comfortable with my body;  I feel anger toward my body;  I like my appearance in spite of its imperfections | Orbach I and Mikulincer M (1998) The body investment scale: construction and validation of a body experience scale. Psychol Assessment 10(4):415-425 |
| FAMILY SOCIAL |  |  |
| Eating together | *How often do you do the following? Please tick one box for each line.* (‘every day’, ‘5-6 days a week’, ‘ 3-4 days a week’, ‘1-2 days a week’, ‘never’)  Have an evening meal together with your mother or father | HBSC survey 2005/06 Optional packag |
| Eating rules | *Here are some statements about eating meals at home. Please say how much you agree or disagree with each one*. (‘strongly agree’, ‘agree’, ‘disagree’, ‘strongly disagree’)  In my family, there are rules at mealtimes that we are expected to follow;  In my family, it is ok for a child to have something else to eat if he/she doesn’t like the food being served;  In my family, a child should eat all the food served even if he/she doesn’t want to;  In my family, manners are important at the dinner table;  In my family, we don’t have to eat all meals at the kitchen or dining room table | Adapted from Neumark-Sztainer D, Wall M, Story M, Fulkerson JA (2004) Are family meal patterns associated with disordered eating behaviours among adolescents? J Adolescent Health 35(5):350-9 |
| Supporting physical activity | *During a typical week how often your parents (or guardian)…* (‘never’, ‘seldom’, ‘sometimes’, ‘often’, ‘very often’)  Encourage you to have physical exercise or sports;  Have physical exercise with you | Finnish HBSC survey |
| General social support | *We are interested in how you feel about the following statements. Read each statement carefully. Indicate how you feel about each statement*. (‘very strongly disagree (1)’, ‘2’, ‘3’, ‘4’, ‘5’, ‘6’, ‘very strongly agree (7)’)  My family really tries to help me;  I get the emotional help and support I need from my family;  I can talk about my problems with my family;  My family is willing to help me make decisions; | Adapted from Zimet G and Grodaon K (1988) The multidimensional scale of perceived social support. J Pers Assess 52(1):30-41 |
| School-related social support | *Here are some statements about your parents. Please show how much you agree or disagree with each one.* (‘strongly agree’, ‘agree’, ‘neither agree nor disagree’, ‘disagree’, ‘strongly disagree’)  If I have a problem at school, my parents are ready to help me;  My parents are willing to come to school to talk to teachers;  My parents encourage me to do well at school;  My parents are interested in what happens to me at school;  My parents are willing to help me with my homework | HBSC Study 2001/02 Optional package |
| Family communication | *How easy is it for you to talk to the following persons about things that really bother you?*  (‘very easy’, ‘easy’, ‘difficult’, ‘very difficult’, ‘don’t have or see this person’)  Father  Stepfather (or mother’s boyfriend)  Mother  Stepmother (or father’s girlfriend) | HBSC surveys 1985/86, 1989/90, 1993/94, 1997/98, 2001/02, 2005/06, 2009/10 |
| Quality of family communication | *In my family…*  (‘strongly agree’, ‘agree’, ‘neither agree nor disagree’, ‘disagree’, ‘strongly disagree’)  I think the important things are talked about  When I speak someone listens to what I say;  We ask questions when we don’t understand each other;  When there is a misunderstanding we talk it over until it’s clear | White MA, Grzankovski J, Paavilainen E, Astedt-Kurki P, Paunonen-llmonen M (2003) Family Dynamics and Child Abuse and Neglect in Three Finish Communities. Issues Ment Health N 24(6-7):707-722  Lasky P, Buckwalter K, Whall A, Lederman R, Speer J, Mc Lane A, King J, White M (1985) Developing an instrument for the assessment of family dynamics. Western J Nurs Res 7:40-57 |
| Parental monitoring | *How much does your mother/father really know about…* (‘she/he knows a lot’, ‘she/he knows a little’, ‘doesn’t know anything’, ‘don’t have or don’t see mother/father’)  Who your friends are;  How you spend your money;  Where you are after school;  Where you go at night;  What you do with your free time;  What you do on the internet | Brown BB, Mounts N, Lamborn SD, Steinberg L (1993) Parenting practices and peer group affiliation in adolescence. Child Dev 64(2):467-82 |
| FRIENDS-SOCIAL |  |  |
| Communication | *How easy is it for you to talk to the following persons about things that really bother you?* (‘very easy’, ‘easy’, ‘difficult’, ‘very difficult’, ‘don’t have or see this person’)  Best friend | HBSC surveys 1985/86, 1989/90, 1993/94, 1997/98, 2001/02, 2005/06, 2009/10 |
| General social support | *We are interested in how you feel about the following statements. Please show how much you agree or disagree with each one.* (‘very strongly disagree (1)’, ‘2’, ‘3’, ‘4’, ‘5’, ‘6’, ‘very strongly agree (7)’)  My friends really try to help me;  I can count on my friends when things go wrong;  I have friends with whom I can share my joys and sorrows;  I can talk about my problems with my friends | Adapted from Zimet G and Grodaon K (1988) The multidimensional scale of perceived social support. Journal Pers Assess 52(1):30-41 |
| Student support | *Here are some statements about the students in your class(es). Please show how much you agree or disagree with each one.* (‘Strongly agree’, ‘agree’, ‘neither agree nor disagree’, ‘disagree’, ‘strongly disagree’)  The students in my class(es) enjoy being together;  Most of the students in my class(es) are kind and helpful;  Other students accept me as I am | HBSC surveys 1993/94, 1997/98, 2001/02, 2005/6, 2009/10  See  Torsheim T, Samdal O, Rasmussen M, Freeman J, Griebler R, Dur W (2010) Cross-National Measurement Invariance of the Teacher and Classmate Support Scale. Soc Indic Res 105(1):145-60 |
| Loneliness | *Do you ever feel lonely?* (‘yes’, very often’, ‘yes, quite often’, ‘yes, sometimes’) | Finnish national HBSC survey |
| SCHOOL-SOCIAL |  |  |
| Teacher support | *Here are some statements about your teachers. Please show how much you agree or disagree with each one.* (‘strongly agree’, ‘agree’, ‘neither agree nor disagree’, ‘disagree’, ‘strongly disagree’)  I feel that my teachers accept me as I am;  I feel that my teachers care me as a person;  I feel a lot of trust in my teachers | Adapted from Torsheim T, Samdal O, Rasmussen M, Freeman J, Griebler R, Dur W (2010) Cross-National Measurement Invariance of the Teacher and Classmate Support Scale. Soc Indic Res 105(1):145-60 |
| School related competence/  autonomy | *We are interested in how you feel about the following statements. Please show how much you agree or disagree with each one.* (‘strongly agree’, ‘agree’, ‘neither agree nor disagree’, ‘disagree’, ‘strongly disagree’)  My teachers encourage me when I do school work;  When I need extra help, I can get it;  My teachers tell me how to do better on school-tasks;  My teachers guide me on how to solve task;  I feel that my teachers provide me with choices and options;  My teachers try to understand how I see things before suggesting a new way to do things;  My teachers make sure I really understand my goals and what I need to do;  My teachers listen to how I would like to do things | HBSC survey 2009/10 Optional package |
| Participation | *Here are some statements about the students in your class(es). Please show how much you agree or disagree with each one* (‘strongly agree’, ‘agree’, ‘neither agree nor disagree’, ‘disagree’, ‘strongly disagree’)  In my classes, students get to participate in deciding class rules;  In my classes, students have some control in deciding which tasks to work on;  In my classes, students get to participate in deciding how to work on tasks | Adapted from Stornes T, Bru E, Idsoe T (2008) Classroom social structure and motivational climates: On the influence of teachers' involvement, teachers' autonomy support and regulation in relation to motivational climates in school classrooms. Scandinavian J Educ Res 52(3):315-29 |
| School perception | *Here are some statements about the students in your class(es).* *Please show how much you agree or disagree with each one. Please tick one box for each line*. (‘strongly agree’, ‘agree’, ‘neither agree nor disagree’, ‘strongly disagree’)  I feel safe in this school;  Pupils are treated in too severe of a manner in our school;  The rules in our school are fair;  Our school is a nice place;  I feel that I belong to this school | HBSC Survey 2001/02 Optional Package |
| HUMAN |  |  |
| Learning difficulties | *Have you had…*(‘no’, ‘some’, ‘noticeable’)  difficulties in reading or spelling  difficulties in mathematics | Finnish national HBSC survey |
| Educational aspiration | *What do you think you will do when you finish compulsory basic*  Try to enter general upper secondary education;  Try to enter vocational upper secondary education or other vocational training ;  Try to get an apprenticeship;  Double examination (e.g. general upper secondary education and vocational upper secondary education);  Get a job;  Be unemployed;  Don’t know | Finnish national HBSC survey |
| Health literacy | *From the following options, choose the one that best describes your opinion.* (‘not at all true’, ‘not completely true’, ‘somewhat true’, ‘absolutely true’)  Having good information on health;  Ability to give example of things that promote health;  Ability to find health-related information that is easy to understand;  Ability to follow the instruction that is easy to understand;  Ability to follow the instructions by doctors and nurses;  Ability to decide if health-related information is right or wrong;  Ability to compare health-related information from different sources;  Ability to justify one’s own choices regarding health;  Ability to determine how one’s own behavior effects one’s health;  Ability to determine how one’s own action effects the surrounding environment;  Ability to give ideas on how to improve health in one’s immediate surroundings  (NOTE: the exact items have not been published) | Paakkari O, Torppa M, Kannas L, Paakkari L (2016) Subjective health literacy: development of a brief instrument for school-aged children. Scand J Public Health 44(8):751-7 |
